# An overview of the history and development of naturopathy in South Africa

**DOI:** 10.4102/hsag.v23i0.1078

**Published:** 2018-07-31

**Authors:** Wendy G. Ericksen-Pereira, Nicolette V. Roman, Rina Swart

**Affiliations:** 1 School of Natural Medicine, Faculty of Community and Health Sciences, University of the Western Cape, South Africa; 2 Child and Family Studies, Faculty of Community and Health Sciences, University of the Western Cape, South Africa; 3 Dietetics and Nutrition, Faculty of Community and Health Sciences, University of the Western Cape, South Africa

## Abstract

**Background:**

A huge growth in complementary and alternative medicine (CAM) took place in South Africa in the 1960s which paralleled what was happening in other parts of the western world. Naturopathy has been practised in South Africa for over 60 years, and the history of naturopathy is entwined with the broader history of CAM. No laws existed at that stage to regulate the curriculum, education and training of CAM practitioners. With the passage of time, various statutes were introduced which eventually led to changes in legislation and the establishment of a recognised training programme. Naturopathy became a legally regulated profession, the full history of which has never been documented.

**Objective:**

This article explores the history of naturopathy in South Africa.

**Method:**

A two-phase qualitative research design was used, consisting of a document search and semi-structured interviews with key informants who were identified through a process of snowballing. Information collected from the naturopaths who participated in the interviews was triangulated with documentation uncovered in the archives of the Allied Health Professions Council of South Africa (AHPCSA) and other literature available.

**Results:**

The result is a history of events which took place and reveals the effect of various legislations on the profession.

**Conclusion:**

Changes in the political system paved the way for changes in legislation which allowed for the registration and training of naturopathic practitioners. However, the lack of a functioning association and the small number of naturopathic graduates have hampered the growth of the profession, preventing it from becoming a significant contributor to the health care system.

## Introduction

The emerging trend is to use the term ‘traditional and complementary medicine (T&CM)’ as it encompasses the practices, practitioners and products of both traditional and complementary medicine (WHO 2013). For the purposes of this research, the term ‘CAM’ has been used as research by Ng et al. (2016) shows that CAM is the most commonly used term to describe complementary medicine. Complementary and alternative medicine (CAM) has been identified as ‘a group of diverse medical and healthcare systems, practices and products that are not presently considered to be part of conventional medicine’ (National Library of Medicine 2010).

The last few decades have seen exponential growth in the use of CAM products and therapies, with Fischer et al. (2014) suggesting that CAM will play an important role in addressing the rise in chronic diseases because of ageing in Europe. The reasons for this shift towards CAM have been proposed as growing disillusionment with the biomedical model of medicine (Reilly 2001), over-prescription of drugs and the impersonal approach to patients within western medicine, together with the inability of the mainstream biomedical model to successfully treat chronic diseases (Chitindingu, George & Gow 2014). South Africa has also experienced a growth in the use of CAM. In 1999 it was estimated that turnover from the use of CAM products was R1.29 billion (Caldis, Mcleod & Smith 2001). In 2014 this figure was estimated to be R8 billion (MCC listens to CAMS concerns 2014; Traditional and Natural Health Alliance 2014).

The history of CAM in South Africa goes back centuries. The early Dutch settlers brought their traditional medicines with them. By the 19th century, there was a small number of CAM practitioners (Gower 2013), but after World War II, South Africa experienced an increase in immigrants, and among them were CAM practitioners. Old Dutch medicines and homeopathic remedies were already in use in the country, but the new wave of immigrants – especially those from Germany – used homeopathy, naturopathy and herbal medicines to treat various illnesses (Pretorius 2010).

Medical practitioners began to campaign against the growing number of CAM practitioners, and this resulted in the Medical Association of South Africa declaring CAM to be ‘illegal and unscientific’ in 1953 (Pretorius 2010:525) and any cooperation between allopathic and CAM practitioners was prohibited in their medical code (Hassim, Haywood & Berger 2007). This meant that CAM practitioners could not share premises with biomedical practitioners, nor refer patients to them. CAM practitioners were therefore excluded from the public health care system. Thus, all CAM modalities were forced into a private health care setting (Pretorius 2010). *The Health Professions Act* 56 (South Africa) 1974 No. 31825 was amended in 2009 with the insertion of rule 8A which states that practitioners are only allowed to share rooms with others who are registered under the Act. Thus, through regulation, all CAM practitioners were legally prevented from working in or making a contribution to the public health care system.

As a system of CAM, the history of naturopathy is reflected in the history of CAM in South Africa. Dr Lilley immigrated to South Africa from the United Kingdom and in 1951 started training the first group of homeopaths (Gower 2013). He was instrumental in establishing Lindlahr College which trained homeopaths, naturopaths and osteopaths (Gower 2013; Prinsloo n.d.). He was one of the founders involved in the formation of the South African Naturopathic and Homeopathic Association (Gower 2013; Prinsloo n.d.); this was the start of the training of naturopaths in South Africa.

Naturopathy in South Africa is defined as a ‘system of healing based on promoting health and treating disease using the body’s inherent biological healing mechanisms to self-heal through the application of non-toxic methods’ (Regulation 127 of 2001). Naturopathic medicine is viewed as a system of primary health care based on the philosophy and principles of naturopathy (Fleming & Gutknecht 2010; Wardle & Oberg 2011). These principles are the healing power of nature, the naturopathic doctor as teacher, finding the root cause of an illness, treating the patient holistically, health promotion and prevention of disease, and encouraging overall wellness (Hausser et al. 2017). As a system of medicine, it is well suited to address the disease challenges of the 21st century as it focuses on preventative medicine through the use of education. By empowering patients to understand the cause of their illness, it encourages a change in lifestyle. Treatment is non-invasive and can be low cost. Naturopaths are well placed to participate in and contribute to the public health care system on a primary health care level. However, at present, the small number of registered naturopathic practitioners, together with the legislature, present a challenge for integration. To understand the current situation, it is necessary to trace the history of naturopathy in South Africa to comprehend how a once burgeoning CAM profession was prevented from training new practitioners for close to 30 years owing to legislative enactments. It is the objective of this article to document the history of naturopathy in South Africa, for it is a story which has never been fully explored.

## Methodology

In order to research the history and development of naturopathy in South Africa, a qualitative design was employed with the research being divided into two phases. The first phase consisted of a document search, while the second phase consisted of interviews with practitioners who were either students during the early years of naturopathy in South Africa or were practising at the time.

### Phase 1: Document search

Permission was obtained from the registrar of the Allied Health Professions Council of South Africa (AHPCSA) to search their archives as per a request for Access to Record of Public Body (section 18[1] of the *Promotion of Access to Information Act* 2 [South Africa] 2000 No. 20852).

Available records were accessed on 3–5 May 2015. The researcher had access to all documents available at the AHPCSA office up to the period that the current registrar took over in 2009, which consisted of over 30 files and record books. Records included registration documents as well as minutes of meetings. It should be noted that there might be gaps in information owing to old documents which had not been accessed for many years by the AHPCSA having been disposed of during a recent move to new premises. Registration records of people deceased or deregistered for longer than 10 years were among the documents not retained, although the original registration applications along with identity document copies were retained. Documentation of all active registrations, regardless of the duration of registration, was also retained.

Documents were separated into the two main themes which emerged. The first theme was registration, as it involved the files of the first practitioners registered as a result of the implementation of the *Homeopaths, Naturopaths, Osteopaths and Herbalists Act* 52 (South Africa) 1974 No. 4441. The second theme was that of minutes of meetings of the different boards within the AHPCSA, starting with the establishment of the first council to regulate natural medicine in South Africa in 1983.

### Phase 2: Interviews

Inclusion criteria for the interviews were determined to include all naturopaths who are currently registered with the AHPCSA or had been registered as a naturopath between the period of 1974 and 2005. Any other registered CAM practitioner who had been registered at any point between 1974 and 1983 was also considered, because of the overlaps in training that took place in the 1960s and 1970s. All naturopaths who graduated from the tertiary training institution after the passing of Regulation 127 of 2001 were excluded.

The researcher went through the list of registered naturopaths on the AHPCSA website and listed all naturopaths with the earliest registration numbers which started with double zero (00). This process was repeated for registered chiropractors, homoeopaths, osteopaths and phytotherapists to establish whether multiple registrations were held. A list was then compiled of the naturopaths who had registered under Act 52 of 1974. By searching the internet – including the Medline directory and the Therapists Online site as well as the telephone directory – the contact details of these early naturopaths were obtained. Contact was made telephonically or via email. The purpose of the communication was explained, and the naturopaths were asked if they were willing to participate in the research project. Five people were identified, of whom four were contacted telephonically. One refused to be interviewed, one agreed to be interviewed, while the receptionists at the practices of the other two advised that the request be sent via email. The fifth person was not contactable via telephone and was emailed. However, none of the emails was responded to. As a result of this low response, a snowball sampling technique was used to identify further participants for the research; this resulted in a total of eight people being interviewed. One person was no longer registered and another was not a naturopath, but, because of their involvement in the 1960s and 1970s, they were interviewed in order to develop a history of CAM in South Africa. Semi-structured interviews were conducted as this allowed the interviewer the opportunity to explore responses in greater detail – this being a necessary consideration, given the age of some of the key informants. Interviews were conducted at the convenience of the participants. Three interviews were conducted face-to-face, three were conducted via skype, one telephonically and one requested the interview questions and provided a written response to the questions via email. All interviews except for one were recorded.

They were then transcribed verbatim, and along with the response received electronically, were thematically coded based on the interview questions and analysed manually (Braun & Clark 2006).

The information sheet, ethics clearance document as well as the interview questions were emailed to the participants before the interview. The identities of the participants were protected through the use of a coding system known only to the researcher. All information gathered in the process of research was securely kept in a password-protected computer and a back-up external hard disk, which, along with other documents collected, is kept under lock and key and is accessible only to the researcher.

Through triangulation of the interviews with the document search and available literature, a history of naturopathy in South Africa was constructed.

## Results

The following themes and sub-themes emerged:

- *Phase 1*: Document Search – registrations and minutes of meetings
- *Phase 2*: Interviews – early history, effect of legislation, activism

### Document search

#### Registrations

Act 52 of 1974 was promulgated in Parliament on 16 October 1974. Section 3(a) of this Act required practitioners to register within a 6-month period from the date of publication of the gazette with the Department of Health. It allowed for the registration of CAM practitioners who were already in practice, provided that they could show proof of their training in the form of a certificate issued by a training institution. Students who were still studying and provided proof of the institution they were studying at were registered as students. Foreign practitioners were in addition required to submit proof of permanent residency or proof that they were ‘capable of acquiring South African citizenship’ (Act 52 0f 1974 3[b i]). Failure to register either as a practitioner or as a student meant that the practitioner would not be allowed to continue practising or the student would not be allowed to register once they had completed their studies.

A summary of practitioners who registered with the Department of Health as a result of the promulgation of Act 52 of 1974 is provided in Table 1.

**TABLE 1:** 1974 practitioner registrations.

Age group: Year born	Naturopathy	Homeopathy	Osteopathy	Herbalism + Homeopathy	Naturopathy + Osteopathy	Naturopathy + Homeopathy	Naturopathy + Herbalism	Homeopathy + Osteopathy	Naturopathy, Homeopathy + Osteopathy	South African	Foreign
Up to 1910	-	5	1	-	1	6	-	1	1	11	4 (American, British + German)
1911–1920	3	18	-	-	-	8	-	4	10	38	6 (British, Cyprian, German, Indian, Irish, Italian)
1921–1930	1	20	-	-	1	17	1	-	11	40	11 (British, Dutch, German, French, Portuguese, Rhodesian)
1931–1940	1	16	1	1	-	12	-	3	14	40	8 (American, Dutch, German, Greek, Indian, Swiss, Portuguese)
1941–1950	-	5	-	-	-	3	-	-	5	12	0
Total	5	64	2	1	2	46	2	8	41	142	29
Percentage	3	37.4	1.2	0.6	1.2	26.9	1.2	4.7	24	83	17

Table 1 is not conclusive as it could not be ascertained if all the applications had been forwarded to the Council by the Department of Health in 1983 or if any of the files were disposed of by the AHPCSA. Gower (2013) puts the number of registered homeopaths at 350. It should be borne in mind that these files only recorded practitioners and not students. The registration process divided applicants into two groups: practitioners and students. Students were allowed to continue with their studies and were registered after 1974 if they had registered as students in 1974.

The data available indicate that the majority of the practitioners who were registered in 1974 were South African, confirming the presence of training institutions in South Africa for each of these professions at the time. It must be noted that multiple registrations predominated, with practitioners registered for homoeopathy and naturopathy being the most common combination (26.9%), followed by homeopathy, naturopathy and osteopathy (23.9%). The various combinations with naturopathy account for 53.2% of registrations, whereas registration for naturopathy alone accounts for only 2.9%. The number of homeopaths who registered only for homoeopathy was 37.4%. From these figures, it can be seen that the number of homeopaths registered were in the majority.

#### Minutes of meetings

The Chiropractors, Homeopaths and Allied Health Service Professions Council became the Allied Health Professions Council of South Africa in 2000 (Caldis et al. 2001; Gower 2013). The Chiropractors, Homeopaths and Allied Health Service Professions Amendment Bill was first published in Government Gazette No. 21825 of 2000 and later promulgated as the *Allied Health Professions Act* 63 (South Africa) R127 2001 No. 22052. This provided for the establishment of professional boards which enabled naturopaths and other diagnostic and therapeutic professions to be registered with their respective professional boards. As no specific system had been used to sort through the files before discarding them, the files which were available were not in chronological order. Minutes of the meeting of the Public Board of Homeopathy, Naturopathy and Phytotherapy (PBHNP) ranged from 2002 to 2005. One of the major issues which appeared most on the agendas of the PBHNP meetings was the registration of naturopaths and phytotherapists. At one PBHNP meeting in 2002, the list of registered naturopaths was tabled. The registration list contained the names, colleges trained at, year of completion of studies and year of first registration as naturopaths. At least 18 naturopathic training colleges existed in the 1960s and 1970s (Appendix 1) with 100 registered naturopaths at the time.

From the minutes of PBHNP meetings held in May 2003, the outcome of applications from naturopaths for registration was tabled. Of the 76 applications, 3 were approved to sit the Council Regulated Examinations (CRE) and 73 were conditionally approved to sit the examination – on condition of submission of proof of a certificate that the naturopathic studies were completed. Not all documents indicated the place of study of the applicants. However, the known institutions of training are summarised in Table 2.

**TABLE 2:** Training institutions.

Name of training institution	Medical doctors	Heilpraktiker (Germany)	Belcher College (USA)	Clayton College of Natural Health (USA)	South African College of Herbal Medicine	South African College of Natural Medicine	South African College of Naturopathic Medicine	Webber Natural Medicine Institute	World Correspondence College
Number of graduates	2	2	2	2	3	8	11	4	5

Of this list of applicants who were conditionally approved to write the CRE, no further minutes were found relating to the number who finally wrote the examination or the outcome of these examinations. However, in minutes tabled in 2003, concerns were raised about the legitimacy of some of the documents that had been submitted.

### Interviews

#### Early history

A detailed history of the early years of naturopathy was developed as a result of the input from all participants. One participant chose to email the response because they did not wish to be recorded and was only prepared to divulge certain information. However, the information which was provided corroborated the information provided by other participants.

The period from the 1950s to 1974 showed rapid growth and training of CAM practitioners in the areas of chiropractic, homeopathy, naturopathy and osteopathy. Private training colleges flourished as there was no control over the registration of these colleges or the curriculum taught. Of the more highly regarded training facilities at the time was the Lindlahr College in Johannesburg. The college offered training in homeopathy, naturopathy and osteopathy (Gower 2013). Naturopaths trained at the Lindlahr College, and records indicate that by 1957, naturopaths were graduating from the college (Prinsloo n.d.). By the 1960s, many training schools of varying quality were flourishing all over the country. One of the interviewees referred to these as the ‘fly by night’ schools.

Interviewees report that many practitioners were trained through ‘apprenticeships’ with other practitioners. Evidence also suggests that there were a number of practitioners from England who either came over for periods of time to teach or settled in the country. In the Cape Town area, Dr Oliver Lawrence, who was a British naturopath, set up a practice at his home, where he taught his students after hours and over weekends. He taught the same curriculum as at Lindlahr College which included subjects such as anatomy, physiology, hygiene theory and homeopathy, among others. Dr Stanley Dean was a herbalist who had a practice on the Foreshore in Cape Town in the late 1960s and he taught the herbal component of the course. All the interviewees agree that there was an abundance of training facilities available at the time. There was a considerable degree of overlap in the training of students in homeopathy, naturopathy, herbal medicine as well as osteopathy; this explains why the early practitioners had a broad range of modalities which they used in practice, and it is the reason for such a high number of dual or multiple registrations (Table 1). On qualification, many of these practitioners went on to establish training centres where they in turn trained other practitioners.

#### Effect of legislation

With the introduction of Act 52 of 1974, all CAM training facilities were to be phased out and shut down. Practitioners were given a period of 6 months to register with the Department of Health. Students who were still training also had to register as students and were allowed to register on completion of their studies. As a result of this Act, hundreds of practitioners were not registered, either because they were not aware of the legislation or because their applications were not approved owing to lack of certification to prove training.

Absence of registration did not stop people from practising, and interviewees confirm that, with the appointment of the first chairperson of the Chiropractors and Homeopaths Association, a serious effort was made to clamp down on unregistered practitioners in practice. If any practitioner was reported to the chairperson, the chairperson reported to the police to follow up. If practitioners were caught in the act, they were arrested and charged with practising unlawfully. This punitive measure did not stop many practitioners who used euphemisms for their practices rather than the term ‘naturopath’, although in essence they still continued to practise as naturopaths. This is evidenced by the number of applicants who applied for registration as a result of the passing of Act 40 of 1995. If there was a lack of certification, it meant they could not write the CRE and were still excluded from the registration process.

*The Associated Health Service Professions Act* 63 (South Africa) 1982 No. 8160 made provision for the establishment of the Associated Health Services Professions Board. As a result of the passing of this Act, all practitioners who had registered in 1974 were required to register again. However, no new registrations were allowed; according to the legislation, there were to be no new registrations of naturopaths after 1982, whereas the register was opened to chiropractors and homeopaths with the establishment of the *Associated Health Service Professions Amendment Act* 105 (South Africa) 1985 No. 9867. This Act also gave the new board the power to control and regulate the education of allied registered practitioners. According to minutes from 1987, training for chiropractors and homeopaths was approved by the Minister of Education in 1987 and courses officially started in 1989 (Chiropractic education in South Africa 1993). No other training institutions for other professions were allowed.

#### Activism

The situation caused dissatisfaction among the other professions and led to the formation of the Confederation of Complementary Health Associations of South Africa (COCHASA). The South African Naturopathic Association (SANA) was formed in the early 1990s and lobbied as one of the members of COCHASA to open the register. In 1994, after the first democratic South African elections, there was a re-examination of laws in the country, including health care laws. This created an opening for COCHASA to lobby the Minister of Health to launch an investigation into the Chiropractors, Homeopaths and Allied Services Professions Council. As a result, the *Chiropractors, Homoeopaths and Allied Health Service Professions Amendment Act* 40 (South Africa) 1995 No. 16643 was enacted which allowed practitioners who had previously not been able to register, to apply for registration. This led to the institution of what was termed the ‘grandfather’ clause which allowed practitioners to undergo a 2-year period of training to upgrade their training to a level determined to be acceptable to the Council. They then wrote a council regulated examination (CRE) and, if they passed, they were registered.

*The Chiropractors, Homeopaths and Allied Health Service Professions Second Amendment Act* 50 (South Africa) 2000 No. 21825 led to the establishment of the Chiropractors, Homeopaths and Allied Health Service Professions Interim Council which operated from 1995 to 2000. Despite these concessions, COCHASA continued to lobby for the opening of the register which would effectively mean that the training of naturopaths and other allied diagnostic professions would become a possibility. Members of COCHASA presented their case in November 2000 to the Parliamentary Portfolio Committee on Health, who subsequently voted for the registers to be opened. This led to the promulgation of Regulation 127 of 2001 which provided for the opening of the register for the diagnostic professions of ayurveda, Chinese medicine and acupuncture, naturopathy, osteopathy, phytotherapy and unani tibb.

SANA members became actively involved with the AHPCSA to determine the scope of practice, and through a comparative analysis of established international training institutions, drew up the curriculum required for the training of naturopaths. They also sought to actively engage with tertiary institutions to establish training facilities for naturopaths and other diagnostic professions which were not offered in South Africa. A School of Natural Medicine which offered training in naturopathy, phytotherapy, traditional Chinese medicine and unani tibb was opened at a tertiary institution in the Western Cape in 2002 and provides currently what is still the only training for these diagnostic professions in South Africa.

## Discussion

This article explores the development of naturopathy as a system of CAM in South Africa. The findings show that the history of naturopathy is closely aligned with the history of CAM in this country. One of the challenges experienced in conducting this research was finding participants who met the inclusion criteria. Naturopaths who either practised or studied between the 1950s and 1974 are now few in number as many are now deceased, or no longer in practice, or were unwilling to speak about this period, especially the period after the passing of Act 52 of 1974. It was found that participants remembered the events that took place but might have forgotten the precise dates when these occurred. Through the process of triangulating, the validity of the information was established (Carter et al. 2014).

The years after the passing of Act 52 in 1974 have had a long-term effect on practitioners. One of the participants chose to respond to the interview questions via email so that the content of the response was controlled by the participant. Others asked questions about confidentiality, and the researcher had to explain in detail the ethical and confidentiality procedures. There still exists a sense of distrust and fear of being identified by peers.

No documentation of the number of practitioners who registered as a result of this Act could be found. The original registration forms were kept by the Department of Health, and with the establishment of Act 63 of 1982, the documentation was sent to the Council. AHPCSA disposed of some of the archived documents. This decision was made on the basis of the legal precept that permits documentation older than 5 years to be destroyed. No record was kept of which documents were disposed of, so there is no exact record of the numbers of practitioners who were registered. Information provided by the interviewees suggests that the number ran into the thousands. One of the shortcomings in this research is the lack of certainty of the exact numbers of practitioners registered in the years leading up to the establishment of the AHPCSA in 2000.

After 1982, the records show that 17 naturopaths were registered between 1984 and 1996. Nine of these had dual registration as homeopaths and naturopaths, two had dual registration as osteopaths and naturopaths, and five were only registered for naturopathy. Missing information on the CRE and registration, discrepancies in registration numbers and years of qualification appear to confirm allegations by some practitioners of selective and preferential deviations from the regulations. The document search supports some of these claims. Act 63 of 1982 made provision for the establishment of the South African Associated Health Service Professions Board (Gower 2013) which had several objectives – the most important was to ‘assist in the promotion and protection of the health of the population of the Republic’ (1[3a]) and to ‘control the registration of persons in respect of any profession and to set standards for the training of intending practitioners’ (1 [3c]). The evidence available appears to suggest that this council failed in fulfilling these objectives. Practitioners were registered with a minimum of training as doctors and allowed to legally practise.

Act 40 of 1995 allowed for the ‘grandfather’ clause which made provision for practitioners who had not previously been registered to undergo a 2-year period of training to upgrade their training before writing a CRE. The qualifications of some of the applicants are of concern because there was no clear indication that they studied any naturopathy courses. There was no minimum criteria set for entry into the CRE and one sees training colleges springing up to provide this training. This undermined the objective of the council to set appropriate standards for the training of practitioners which would safeguard the health of the public.

According to some of the interviewees, it was after the establishment of the naturopathy training course in 2002 that SANA gradually became less functional. By the time naturopathic students graduated, they found themselves with no functional association able to support and guide their professional development, which has had an adverse effect on the growth of the profession. Drawing from the North American situation, one finds organisations such as the Canadian Association of Naturopathic Doctors actively promoting and advocating for the professionalisation of, and regulatory changes for, naturopathy (National Associations for Naturopathic Doctors n.d.). This activism has resulted in an increase in the number of naturopathic students. In 2009, this advocacy resulted in registered naturopaths being granted the right to prescribe certain categories of pharmaceuticals (Eggertson 2010) which essentially placed them on the same level as general practitioners. In 2016, naturopathic graduates become actively involved in relaunching SANA; this may herald the start of another chapter in the history of naturopathy in South Africa.

## Conclusion

The history of naturopathy in South Africa has not been recorded prior to this article. This research has revealed a period of rapid growth of CAM in the period from 1950s to 1974 which was abruptly halted through the introduction of legislation. As a direct result of continued activism by naturopaths and other CAM practitioners, legislation was changed and has led to legal recognition. This was a time of huge optimism for the CAM sector as it saw the legitimisation of CAM professions through the establishment of the AHPCSA as the start of a period of growth for these professions. This in turn resulted in establishing training programmes at tertiary institutions.

However, this has not resulted in an increase in numbers of registered naturopaths. If naturopaths are to make a significant contribution to the health care system, there has to be a substantial increase in the number of naturopathic graduates as well as a strong association to promote public awareness of naturopathy. Growth and professionalisation of the profession should ultimately lead to a change in legislation.

## Appendix 1

**Table:** Details of 2002 registration:

Year of registration	No registered	Year of completion of studies	College’s qualification was obtained at
1980	1	1972	Naturopathic College of South Africa
1982	22	1954, 57, 58, 61, 62, 63, 64, 1967, 1969, 1970 (x2), 1972, 1973 (x2), 1974 (x4), 1976, 1980	Lindlahr College, Canyon College, College of Natural Sciences, Naturopathic College of South Africa, Lindstrom, South African Faculty of Naturopathy and Osteopathy
1983	6	1969, 1974, 1972, 1974 (x2), 1975	Canyon College, College of South Africa, Lindlahr College, Naturopathic Association, South African Foundation of Natural Therapies
1984	5	1967, 1972, 1974	Lindlahr College, South African Faculty of Homeopathic Medicine
1985	8	1965, 1972, 1974	College of South Africa, Naturopathic Association of South Africa, South African Faculty of Homeopathic Medicine
1987	1	1967	Lindlahr College
1990	2	1973, 1975	College of Natural Sciences, South African Institute of Homeopathy
1993	1	1974	Canyon College
1996	23	1957, 1971, 1973 (x2), 1974 (x7), 1976, 1982, 1989, 1993 (X7)	African Herbal College, Brantridge Forest School, Transkei, Lindlahr College, South African Foundation of Natural Therapies, South African Homeopathic Association, World Correspondence College
1997	7	1973, 1974, 1995	College of Natural Therapy, College of Natural Science, Lindlahr College, Naturopathic College of South Africa
1999	2	1978, 1981	Naturopathic College of South Africa, Brantridge Forest School
2000	1	1972	Naturopathic College of South Africa
2001	3	1973, 1991	Lindlahr College, South African College of Herbalism

Note: The number of registered Naturopaths totalled 100. However, not all on the registration list recorded the year of completion of training or the institute at which the training was undertaken.

## References

[CIT0001] Braun V. & Clark V., 2006, ‘Using thematic analysis in psychology’, *Qualitative Research in Psychology* 3(2), 77–101. 10.1191/1478088706qp063oa

[CIT0002] Caldis K.S., McLeod H.D. & Smith P.R., 2001, ‘The fall of the Bamboo Curtain: A review of complementary medicine in South Africa’, *South African Actuarial Journal* 1, 63–93. 10.4314/saaj.v1i1.24491

[CIT0003] Carter N., Bryant-Lukosius D., DiCenso A., Blythe J. & Neville A.J., 2014, ‘The use of triangulation in qualitative research’, *Oncology Nursing Forum* 41(5), 545–547. 10.1188/14.ONF 25158659

[CIT0004] Chiropractic education in South Africa, 1993, *Dynamic Chiropractic*, 11(18), viewed 24 March 2018, from http://www.dynamicchiropractic.com/mpacms/dc/article.php?id=42496

[CIT0005] Chitindingu E., George G. & Gow J., 2014, ‘A review of the integration of traditional complementary and alternative medicine into the curriculum of South African medical schools’, *Biomed Central Medical Education* 14, 40 10.1186/1472-6920-14-40 24575843 PMC3939811

[CIT0006] Eggertson L., 2010, ‘Naturopathic doctors gaining new powers’, *Canadian Medical Association* 182(1), 109–3112. 10.1503/cmaj PMC2802635 19917661

[CIT0007] Fischer F.H., Lewith G., Witt C.M., Linde K., von Ammon K., Cardini F. et al., 2014, ‘High prevalence but limited evidence in complementary and alternative medicine: Guidelines for future research’, *BMC Complementary and Alternative Medicine* 14, 46. 10.1186/1472-6882-14-46 PMC3931324 24499316

[CIT0008] Fleming S.A. & Gutknecht N.C., 2010, ‘Naturopathy and the primary care practice’, *Primary Care: Clinics in Office Practice* 37(1), 119–136. 10.1016/j.pop.2009.09.002 20189002 PMC2883816

[CIT0009] Gower N., 2013, *A brief look at the history of homeopathy in South Africa: Homeopathy papers past and present*, viewed 25 July 2016, from http://hpathy.com/homeopathy-south-africa/

[CIT0010] Hassim A., Haywood M. & Berger J., 2007, *Health and Democracy: A guide to human rights, health law and policy in post-apartheid South Africa*, Siber Ink CC, Cape Town.

[CIT0011] Hausser T., Lloyd I., Yanez J., Cottingham P., Turner R.N. & Abascal A., 2017, *WNF white paper: Naturopathic philosophies, principles and theories*, viewed 02 February 2018, from www.worldnaturopathicfederation.org

[CIT0012] MCC listens to CAMS concerns, 2014, *Medical Chronicle*, viewed 08 December 2016, from http://www.medicalchronicle.co.za/mcc-listens-cams-concerns/

[CIT0013] National Associations for Naturopathic Doctors (n.d.), *The Canadian Association of Naturopathic Doctors*, viewed 19 December 2016, from https://aanmc.org/national-associations/

[CIT0014] National Library of Medicine, 2010, *Definition of CAM*, viewed 23 March 2018, from https://www.nlm.nih.gov/tsd/acquisitions/cdm/subjects24.html

[CIT0015] Ng J.Y., Boon H.S., Thompson A.K. & Whitehead C.R., 2016, ‘Making sense of “alternative”, “complementary”, “unconventional”, and “integrative medicine”: Exploring the terms and meanings through textual analysis’, *BMC Complementary and Alternative Medicine* 16, 134 10.1186/s12906-016-1111-3 27206976 PMC4875612

[CIT0016] Pretorius E., 2010, ‘Complementary/alternative and traditional healthcare in South Africa’, in van Rensburg H.C.J. (ed.), *Health and Healthcare in South Africa*, pp. 506–560, Van Schaik, Pretoria.

[CIT0017] Prinsloo J.P., (n.d). *History of homeopathic training in South Arica*, viewed 21 March 2018, from http://www.biocura.co.za/homeopathy/history_homeopathic_education_in_south_africa.html

[CIT0018] Reilly D., 2001, ‘Comments on complementary and alternative medicine in Europe’, *Journal of Alternative and Complementary Medicine* 7(Suppl), 23–31. 10.1089/107555301753393779 11822632

[CIT0019] Traditional and National Health Alliance, 2014, *Your right to choose natural health products is under threat*, viewed 28 July 2016, from www.naturalhealthalliance.co.za/DraftAmendmentCommentDec2014.htm

[CIT0020] Wardle J. & Oberg E., 2011, ‘The intersecting paradigms of naturopathic medicine and public health: Opportunities for naturopathic medicine’, *Journal of Alternative and Complementary Medicine* 17(11), 1079–1084. 10.1089/acm.2010.0830 22070439

[CIT0021] World Health Organization 2013, *WHO traditional medicine strategy: 2014–2023*, Geneva, viewed 26 March 2018, from http://www.who.int/medicines/publications/trmstrategy1423/e

